# Integrative Single-Cell and Spatial Transcriptomic Analyses Link Arachidonic Acid Metabolic Reprogramming to Proneural-to-Mesenchymal State Transition in Glioblastoma

**DOI:** 10.3390/biomedicines14081738

**Published:** 2026-08-01

**Authors:** Shiyun Peng, Zhenkun Wen, Xiao-Ting Cai

**Affiliations:** 1 The Second Clinical Medical College, Southern Medical University, Guangzhou 510515, China; 2 The First Clinical Medical College, Southern Medical University, Guangzhou 510515, China

**Keywords:** glioblastoma, drug repurposing, mesenchymal transition, scRNA-seq, stRNA-seq

## Abstract

**Background/Objectives:** Glioblastoma (GBM) is widely recognised as a highly aggressive form of malignant tumour arising within the central nervous system. The proneural-to-mesenchymal (PN–MES) state transition is a key process underlying malignant progression and therapeutic resistance. The present study was designed to elucidate the association between the PN–MES transition and arachidonic acid (AA) metabolic reprogramming in GBM. **Methods:** We integrated four public single-cell RNA-sequencing cohorts, two spatial transcriptomic cohorts, and three bulk RNA-sequencing cohorts for GBM. By combining single-cell transcriptomics, spatial transcriptomics, pseudotime trajectory inference, and cell–cell communication analysis, we systematically evaluated the cell-state dependence and spatial heterogeneity of AA metabolism in GBM. We further incorporated machine learning-based survival modelling, molecular docking, molecular dynamics simulations, and in vitro functional assays to identify and validate potential prognostic biomarkers and therapeutic targets. **Results:** Mesenchymal-like (MES-like) tumour cells showed the highest transcriptionally inferred AA metabolism-related score, and AA metabolism-related gene expression was closely coupled with PN–MES state transition. The peptidylprolyl isomerase A (*PPIA*)–basigin (*BSG*) signalling axis was selectively enriched in tumour cells with high expression of arachidonic acid metabolism-related genes (AAMGs), whereas virtual knockout of *BSG* perturbed MES- and invasion-related gene modules. Spatial transcriptomic analysis confirmed that *PPIA*–*BSG*-associated communication was enhanced within MES-like tumour niches and was coupled with inflammatory and hypoxic programmes. AA metabolism-related genes enabled robust prognostic stratification. Molecular simulation and in vitro experiments suggested that Venetoclax could bind *BSG* and suppress GBM cell viability. **Conclusions:** AA metabolism-related transcriptional reprogramming defines mesenchymal-associated malignant niches in GBM and may contribute to PN–MES-related state evolution through *PPIA*–*BSG*-mediated microenvironmental communication. AA metabolism-related features and the *BSG*-associated signalling axis may provide candidate directions for prognostic stratification and targeted intervention.

## 1. Introduction

Glioblastoma (GBM) represents roughly 14.5% of all central nervous system tumours, constitutes about 48.6% of malignant cases within this category, and remains one of the most aggressive human malignancies [1]. Among the molecular and cellular states of GBM, the mesenchymal subtype is generally associated with enhanced invasiveness and the poorest clinical outcomes. The proneural-to-mesenchymal transition (PN–MES transition) is increasingly recognised as a key biological process driving malignant progression and therapeutic resistance [2,3]. Previous studies have suggested that the PN–MES transition is not governed by a single molecular event, but shaped by multiple convergent pressures. For example, tumour microenvironmental cues can activate NF-κB signalling in tumour cells, thereby promoting their shift towards a mesenchymal-like phenotype [4,5,6]. Therapeutic stressors, including radiotherapy and temozolomide chemotherapy, may also induce PN–MES transition, and the acquired mesenchymal phenotype can further confer resistance to multiple treatment modalities [7,8]. In parallel, transcriptional reprogramming driven by regulators such as STAT3, C/EBPβ, and FOSL1 has provided important mechanistic clues for understanding the PN–MES transition in GBM [9,10,11].

To sustain their highly proliferative and adaptive phenotype, GBM cells must overcome fluctuations in nutrient and energy availability imposed by a heterogeneous tumour microenvironment [12]. Metabolic reprogramming is therefore central to GBM progression. Arachidonic acid (AA) metabolism, mediated through the three canonical cyclooxygenase, lipoxygenase, and cytochrome P450 branches and their downstream lipid mediators, regulates a wide range of pathophysiological processes, including tumour cell proliferation, angiogenesis, inflammation, and stress adaptation [13,14,15]. These features suggest that AA metabolism may participate in the mesenchymal evolution of GBM; however, its specific role in the PN–MES transition remains insufficiently defined [16]. Previous lipidomic studies in glioma have contributed to molecular subtype classification, prognostic modelling, and the discovery of early diagnostic biomarkers [17,18,19]. Nevertheless, emerging spatial transcriptomic evidence indicates that metabolic reprogramming in glioma is highly spatially heterogeneous and closely associated with the cellular composition and intercellular interactions of specific tumour niches [12,20,21].

Traditional bulk-level analyses are unable to resolve the spatial heterogeneity of cell-state transition, metabolic reprogramming, and microenvironmental crosstalk in GBM. A multi-omics integrative strategy is therefore required. In the present study, we brought together single-cell, spatial, and bulk transcriptomic datasets and further incorporated pseudotime analysis, cell–cell communication inference, and molecular docking, along with in vitro experiments to support the analysis. Our findings suggest that MES-like GBM cells may influence AA metabolism through the peptidylprolyl isomerase A (*PPIA*)–basigin (*BSG*) axis and NF-κB-associated signalling, thereby linking metabolic reprogramming with malignant cell-state transition. We further identified candidate prognostic markers and therapeutic candidates, providing a framework for understanding AA metabolism-associated malignant niches in GBM.

## 2. Materials and Methods

### 2.1. Data Sources

Single-cell RNA-sequencing data for GBM were retrieved from the Gene Expression Omnibus database (GEO; http://www.ncbi.nlm.nih.gov/geo/ accessed on 27 March 2026), including GSE103224, GSE138794, GSE139448, and GSE230389. Spatial transcriptomic data were retrieved from two publicly available Visium cohorts, GSE194329 and GSE235672. Transcriptomic profiles and clinical follow-up information for the TCGA-GBM cohort were acquired from the UCSC Xena platform (https://xena.ucsc.edu/). For external validation, gene expression datasets were collected from the GEO cohorts GSE7696 and GSE83300. Arachidonic acid metabolism-related genes (AAMGs) were derived from the KEGG pathway hsa00590. Available sample-level annotations, including *IDH* status, original molecular subtype when reported, *EGFR* status, and other genomic or transcriptomic features were summarised in Supplementary Table S1.

### 2.2. Single-Cell RNA-Sequencing Data Processing

Single-cell RNA-sequencing data were analysed using Scanpy version 1.11.5, with deep generative modelling performed via scvi-tools, version 1.4.2, within the scVI framework. To retain high-quality cellular transcriptomes, genes expressed in at least three cells were included. Cells were filtered out if they contained fewer than 200 or more than 8000 detected genes. In addition, those exhibiting over 20% mitochondrial transcripts or exceeding 50% ribosomal gene content were also removed from downstream analyses. Potential doublets were removed using doubletdetection, version 4.3 [22].

After integration, the expression matrix was normalised by total counts and log1p-transformed. Using sample identity as the batch key, a total of 2000 genes exhibiting the highest variability were retained for subsequent analysis. The raw count layer was then used as input to construct an SCVI model for batch correction and latent representation learning. Based on the X_scVI latent space, a nearest-neighbour graph was first established; clustering was then carried out using Leiden algorithm with a resolution parameter set to 0.8. UMAP visualisation was initialised using PAGA. Cell identities were assigned manually based on established canonical marker genes.

### 2.3. Inference of Copy Number Variation

Copy number variation was inferred from raw single-cell transcriptomic count matrices using the inferCNV R package, version 1.26.0. Cell annotation information and gene genomic position files were used to construct the inferCNV object, with all cells not annotated as tumour cells defined as normal reference cells. inferCNV was then run with a cutoff value of 0.1 to identify chromosomal amplification and deletion patterns, thereby assisting in the discrimination of malignant cells.

### 2.4. Trajectory Analysis

Trajectory inference and pseudotime analysis of malignant cells from GBM samples were performed using Monocle3, version 1.4.26 [23], to characterise dynamic cell-state transitions and evaluate the distribution of malignant subpopulations along the inferred trajectory. A cell_data_set object was first generated using the expression matrix together with cell and gene annotation information. Batch effects were removed using the align_cds function, followed by dimensionality reduction and clustering based on UMAP.

The cellular developmental trajectory was subsequently inferred using the learn_graph function, followed by pseudotime ordering of cells with the order_cells function. Finally, trajectory visualisation was performed using the built-in Monocle3 plotting functions. Dynamic expression patterns of metabolism-related genes along pseudotime were further visualised as heatmaps using ClusterGVis, version 0.99.9 [24]. To contextualise upstream PUFA processing, we additionally examined linoleic acid metabolism-related genes from KEGG hsa00591 and representative fatty acid uptake/desaturation/elongation genes, including *CD36*, *FADS1*, *FADS2*, and *ELOVL5*, along the same pseudotime axis.

### 2.5. Cell–Cell Communication Analysis

Cell–cell communication patterns within the scRNA-seq dataset were investigated using CellChat, version 2.2.0 [25]. CellChat objects were generated using the gene expression matrix together with the corresponding cell type annotation data. CellChatDB.human was used as the ligand–receptor reference database. Overexpressed ligand–receptor pairs were identified, and intercellular communication probabilities were calculated according to the standard CellChat workflow.

Communication events involving cell populations with fewer than ten cells were filtered out. Communication networks were then inferred and aggregated at the signalling-pathway level. Finally, communication patterns between AAMGs-high or AAMGs-low tumour cells and other cellular populations were compared.

### 2.6. Virtual Knockout Analysis

To evaluate the transcriptomic consequences of *BSG* perturbation, we performed an in silico virtual knockout analysis using the machine learning-based scTenifoldKnk framework, which is specifically designed for single-cell RNA-sequencing data [26]. To further explore the functional associations among genes affected by scTenifoldKnk-based virtual knockout, a protein–protein interaction network was constructed based on the STRING database and subsequently visualised using Cytoscape, version 3.10.4 [27,28]. *BSG* virtual knockout was restricted to the MES-like malignant cell subset to better align with the focus and rationale of our study.

Module detection within the PPI network was carried out by means of MCODE, version 2.0.3, to identify densely connected functional subnetworks [29]. CytoHubba, version 0.1, was subsequently utilised to compute topological metrics for network nodes and to identify candidate hub genes [30].

### 2.7. Cell2location-Based Deconvolution of Spatial Transcriptomes

Cell2location, version 0.1.5, was used to deconvolve spatial transcriptomic data by projecting single-cell transcriptomic signatures onto spatial transcriptomic profiles [31]. The model was run for 30,000 iterations to achieve convergence and to estimate the probability distribution of cell type composition across spatial spots.

Through integration of the scRNA-seq reference with spatial transcriptomic data, the model efficiently mitigated technical variability while maintaining intrinsic biological signals, thereby facilitating high-resolution delineation of cell type-specific spatial distributions within tumour tissues.

### 2.8. Pathway Activity Inference and Spatial Interaction Analysis

To explore spatially resolved intercellular interactions, we used COMMOT, version 0.0.3, to infer spatial communication based on ligand–receptor expression and spatial coordinates [32]. The analysis used CellChatDB as the ligand–receptor reference and focused on the CypA/*PPIA*–*BSG* signalling axis across multiple spatial transcriptomic samples. Communication directionality, inter-cluster communication networks, and associated downstream gene changes were evaluated.

The results from COMMOT were analysed across datasets to determine reproducible communication patterns and potential tissue-specific differences. Pathway activity inference was performed using the decoupler package in Python, version 2.1.4. Based on the human PROGENy model, pathway activity scores for each spatial location were calculated using the univariate linear model method.

### 2.9. Machine Learning Prognostic Modelling

To assess the prognostic value of arachidonic acid metabolism-related genes, the TCGA-GBM cohort served as the training set, whereas the GSE83300 and GSE7696 cohorts were used as external validation sets. We used 63 arachidonic acid metabolism-related genes as candidate features for survival model construction.

Prognostic prediction models were constructed and subsequently evaluated in comparison using the R package Mime1, version 0.0.0.9000 [33,34], which provides an integrated framework of ten different machine learning algorithms for survival analysis. A total of 101 algorithm combinations were evaluated, and the StepCox[both] + RSF model was selected as the optimal model based on overall predictive performance. The final model was applied to compute an individual risk score for each patient, and patients in each cohort were allocated to high-risk and low-risk groups according to the median risk score. Differences in overall survival between the two groups were evaluated by constructing Kaplan–Meier survival curves.

### 2.10. Molecular Docking

Molecular docking simulations were carried out with AutoDock Vina, version 1.2.7 [35,36]. The receptor structure was based on the AlphaFold-predicted *BSG* protein model. The docking box was centred at x = 2.730, y = 20.750, and z = 8.270, with the box dimensions set to 20.0 Å along the *x*, *y*, and *z* axes.

After docking, ligand conformations were ranked according to the Vina binding affinity score. Hydrogen bonds and hydrophobic interactions between the receptor and ligand were visualised and analysed using PyMOL, version 3.1.7.2.

### 2.11. Molecular Dynamics Simulation

Molecular dynamics simulations were performed using GROMACS, version 2025.4 [37,38]. The protein was parameterised using the CHARMM36m force field, and ligand parameters were generated using CGenFF, version 5.0 [39,40,41]. After solvation and ion neutralisation, the system first underwent energy minimisation, after which it was equilibrated under NVT ensemble conditions for 1 ns, followed by a 2 ns equilibration under NPT ensemble at 300 K. Production simulations were then performed for 50 ns with a time step of 2 fs. The structural stability of the protein–ligand complex was evaluated on the basis of root-mean-square deviation, root-mean-square fluctuation, and radius of gyration analyses.

### 2.12. Cell Culture

U87, LN229, A172, and U251 human glioblastoma cell lines were obtained from the Cell Bank of the Chinese Academy of Sciences (Shanghai, China). The cells were maintained in Dulbecco’s modified Eagle medium (DMEM; Gibco, Thermo Fisher Scientific, Waltham, MA, USA) supplemented with 10% foetal bovine serum (FBS; Gibco, Thermo Fisher Scientific, Waltham, MA, USA) and 1% penicillin–streptomycin (Wuhan Servicebio Technology Co., Ltd., Wuhan, China). Cell cultures were incubated at 37 °C in a humidified atmosphere supplemented with 5% CO_2_. The culture medium was replaced every 2 days. Cells at the exponential growth stage were selected for subsequent drug exposure and functional analyses.

### 2.13. Cytotoxicity Assay and U251-Based Validation Experiment

Cell viability was assessed using the Cell Counting Kit-8 assay (CCK-8; Wuhan Servicebio Technology Co., Ltd., Wuhan, China). Cells from the U87, LN229, and A172 lines were distributed into 96-well plates, with 1 × 10^4^ cells seeded in each well. After culturing in serum-free medium for 24 h, cells were exposed to Venetoclax (TargetMol Chemicals Inc., Wellesley Hills, MA, USA) at concentrations ranging from 0.1 μM to 100 μM (0.1, 0.5, 1, 5, 10, 50, and 100 μM) for a duration of 48 h.

The culture medium was subsequently replaced with fresh medium, and 10 μL of CCK-8 reagent was added to each well. The cells were then incubated at 37 °C for 2 h. Measurements of absorbance at 450 nm were obtained using a microplate reader to evaluate changes in cell viability under different treatment conditions.

U251 cells were also used for a PN–MES-related validation experiment using a conventional GBM cell line model. For the U251 validation, cells were seeded in 96-well plates and assigned to four treatment groups: DMSO (Thermo Fisher Scientific Inc., Waltham, MA, USA), TGF-β1 (TargetMol Chemicals Inc., Wellesley Hills, MA, USA), Venetoclax, or TGF-β1 plus Venetoclax. After 48 h of treatment, CCK-8 absorbance at 450 nm was measured using the procedure described above, with each condition measured in triplicate. Parallel cultures were analysed by Western blotting for N-cadherin and CD44 expression. This cell line model was selected because previous studies showed that activation of the TGF-β/TGF-β1 pathway can induce mesenchymal-like, PN–MES-related phenotypic changes in U251/U251 MG glioma cells, including increased N-cadherin and CD44/stemness-related features and migration/invasion through SMAD2/3-, Akt-, Erk-, or TGFβ1/Smad-related signalling [42,43,44,45,46].

### 2.14. Western Blotting

Total cellular proteins were extracted using RIPA lysis buffer (cat. no. G2002; Wuhan Servicebio Technology Co., Ltd., Wuhan, China) supplemented with protease and phosphatase inhibitors (cat. no. G2006 and G2007; Wuhan Servicebio Technology Co., Ltd., Wuhan, China). A BCA protein assay kit (cat. no. G2026; Wuhan Servicebio Technology Co., Ltd., Wuhan, China) was employed to quantify protein concentration, after which the samples were mixed with loading buffer and boiled for 10 min before further processing.

A total of 10 μg protein per lane was electrophoresed by SDS–PAGE (10% resolving, 5% stacking gels) and then transferred to polyvinylidene fluoride membranes. Following blocking with 5% non-fat milk at room temperature for 2 h, the membranes were subsequently incubated with primary antibodies overnight at 4 °C. After overnight incubation, the membranes were rinsed three times in TBST buffer and subsequently exposed to secondary antibodies for 2 h at room temperature. Immunoreactive bands were detected by enhanced chemiluminescence and recorded using a GS-700 imaging densitometer (Bio-Rad Laboratories, Inc., Hercules, CA, USA). The resulting images were used for protein band quantification.

### 2.15. Statistical Analysis

Most statistical computations were performed using R (4.5.2) and Python (3.9), with appropriate packages. The comparison of overall survival between high- and low-risk groups in both cohorts was performed using Kaplan–Meier survival analysis with the log-rank test. The predictive performance of the prognostic model was primarily assessed using the concordance index.

Continuous variables between the two groups were evaluated using the non-parametric Wilcoxon rank-sum test. Correlation analyses were performed using Spearman’s rank correlation test. Spatial autocorrelation was assessed using Moran’s I statistic. All analyses were conducted using two-tailed statistical tests, with a *p* value below 0.05 or an FDR less than 0.05 regarded as indicative of statistical significance.

## 3. Results

### 3.1. Integrated Single-Cell Transcriptomic Analysis Reveals the Cellular Landscape and Malignant-State Heterogeneity of GBM

**Figure 1:**
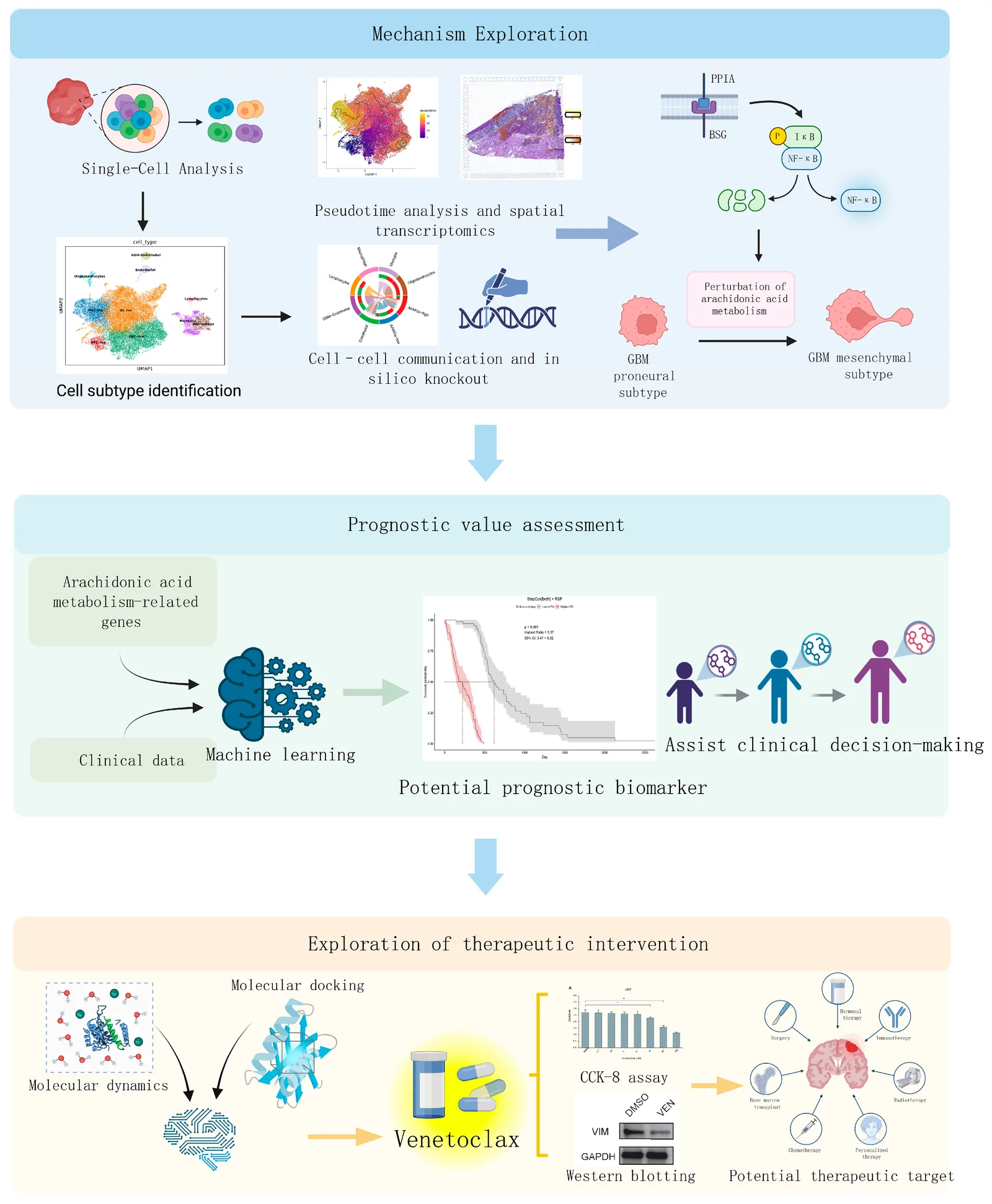
Flow chart of this study. * *p* < 0.05; ** *p* < 0.01.

An overview of the study design is presented schematically in Figure 1. We integrated four publicly available GBM single-cell transcriptomic cohorts, including GSE103224, GSE138794, GSE139448, and GSE230389. After standardised quality control, 35,374 cells from 11 patients were retained for downstream analysis.

Following scVI-based batch correction and marker-gene-based annotation, ten major cellular populations were identified. These included macrophages, marked by *CD68* and *CD163*, accounting for 3.52% of all cells; microglia, marked by *CX3CR1* and *TMEM119*, accounting for 4.35%; lymphocytes, marked by *CD3D* and *NKG7*, accounting for 0.78%; endothelial cells, marked by *VWF* and *PECAM1*, accounting for 1.15%; GBM-associated endothelial cells, marked by *COL1A2* and *RGS5*, accounting for 0.83%; and oligodendrocytes, marked by *MOG* and *MAG*, accounting for 0.98%.

**Figure 2:**
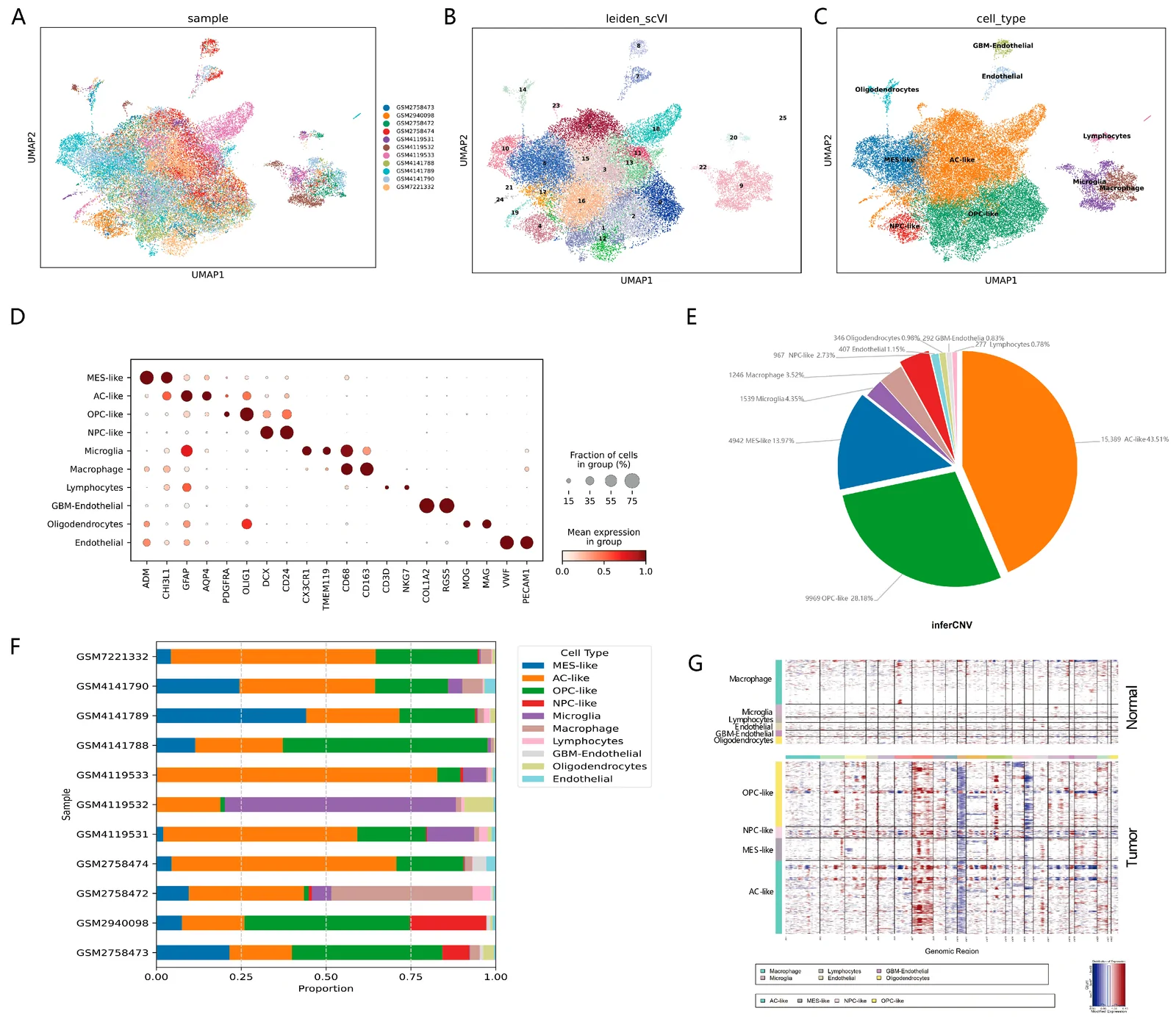
(**A**–**C**) UMAP visualisation of 35,374 cells integrated from four GBM single-cell RNA-seq datasets, coloured according to sample origin, Leiden clustering, and annotated cell identity, respectively. Ten major cellular populations were identified, including MES-like, AC-like, OPC-like, and NPC-like malignant cells, as well as microglia, macrophages, lymphocytes, endothelial cells, GBM-associated endothelial cells, and oligodendrocytes. (**D**) Dot plot was used to visualise the expression profiles of representative marker genes across the annotated cell populations. In this representation, the dot size corresponds to the fraction of cells expressing a given gene, while the colour intensity reflects its mean expression level. (**E**) Pie chart summarising the overall proportions of each cell type in the integrated GBM single-cell dataset. (**F**) Stacked bar plot displaying the relative abundance of different cell populations across individual patient samples, highlighting marked inter-sample heterogeneity in cellular composition. (**G**) Copy number variation profiles inferred by inferCNV, showing relative chromosomal alterations in tumour cells compared with non-malignant reference cells; red indicates copy number gain, and blue indicates copy number loss, supporting the identification of malignant cell populations.

Four malignant cellular states were further identified, including MES-like cells, marked by *ADM* and *CHI3L1*, accounting for 13.97%; AC-like cells, marked by *GFAP* and *AQP4*, accounting for 43.50%; OPC-like cells, marked by *PDGFRA* and *OLIG1*, accounting for 28.18%; and NPC-like cells, marked by *DCX* and *CD24*, accounting for 2.73% (Figure 2A–E). The composition of malignant cells varied markedly across patients (Figure 2F), underscoring the extensive inter-patient heterogeneity of GBM. In addition, inferCNV analysis revealed recurrent chromosome 7 amplification and chromosome 10 deletion in tumour cells (Figure 2G), supporting the reliability of cell annotation and malignant cell identification.

### 3.2. Arachidonic Acid Metabolism Is Enriched in MES-like Cells and Associated with PN–MES State Transition

**Figure 3:**
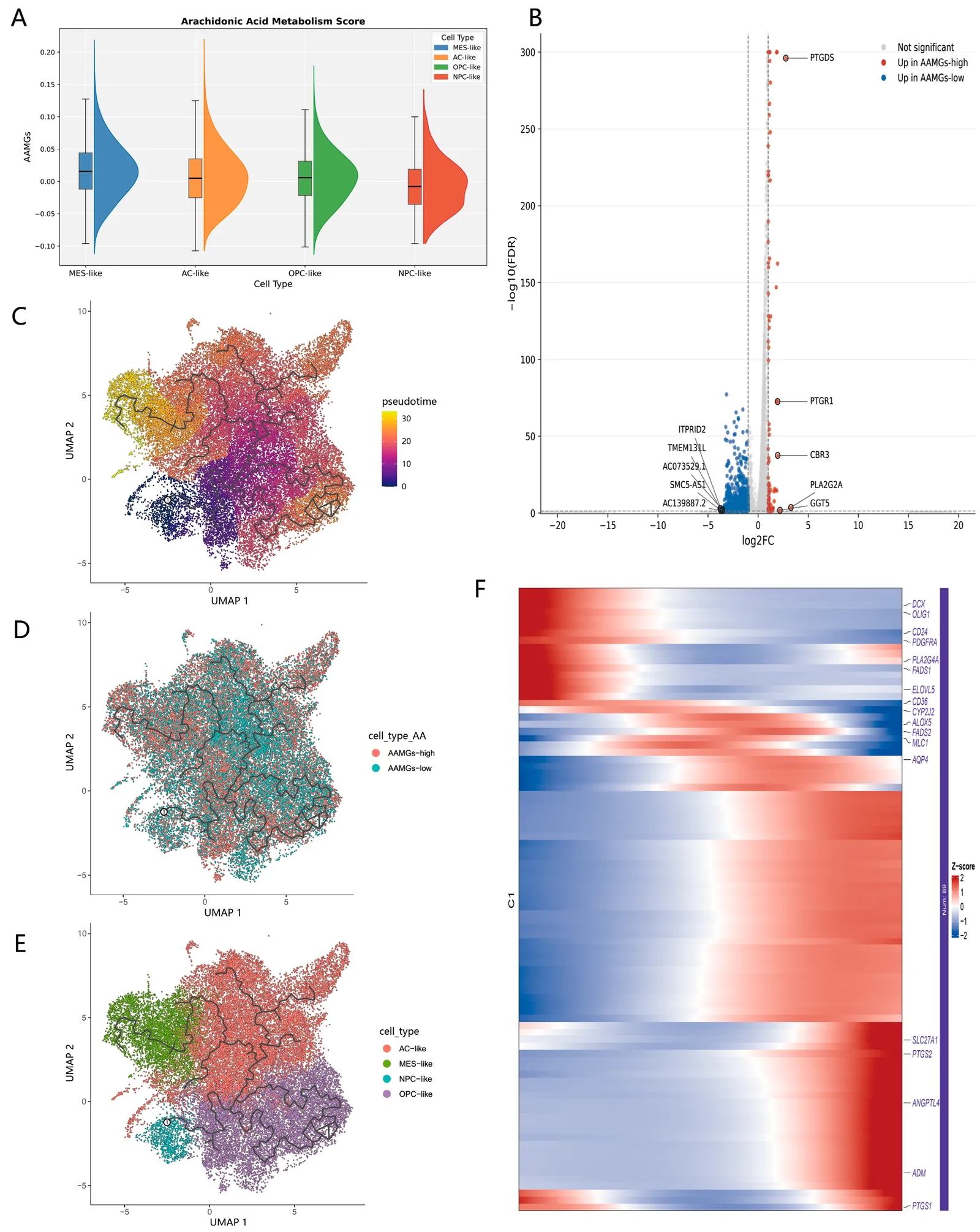
Distribution and dynamic changes of arachidonic acid metabolic activity during malignant cell-state transition in GBM. (**A**) Violin plots show arachidonic acid metabolism-related gene scores across malignant cell subpopulations, with MES-like cells displaying relatively higher metabolic activity. (**B**) Volcano plot showing differentially expressed genes between AAMGs-high and AAMGs-low malignant cells; red indicates genes upregulated in the AAMGs-high group, blue indicates genes upregulated in the AAMGs-low group, and grey indicates non-significant genes. (**C**) Monocle3-based pseudotime analysis showing the state progression of malignant cells in UMAP space. (**D**) Distribution of AAMGs-high and AAMGs-low malignant cells along the pseudotime trajectory, suggesting a transition from an earlier AAMGs-low state toward an AAMGs-high state. (**E**) Localisation of AC-like, MES-like, NPC-like, and OPC-like malignant cell states within the same trajectory space, indicating progression from earlier NPC-like/OPC-like states toward AC-like/MES-like states. (**F**) Heatmap showing pseudotime-dependent expression patterns of representative genes, including arachidonic acid metabolism-related genes, upstream PUFA-processing genes, and cell-state marker genes; red indicates relatively high expression, and blue indicates relatively low expression.

Using 63 arachidonic acid metabolic genes derived from the KEGG hsa00590 pathway, we calculated module scores for malignant cells and found that the transcriptionally inferred AA metabolism-related score exhibited marked cell-state dependence. Among the malignant populations, MES-like cells showed the highest AA metabolism-related score (Figure 3A).

Malignant cells were then stratified into AAMGs-high and AAMGs-low groups according to the median score of arachidonic acid metabolic genes. Differential expression analysis revealed broad transcriptomic differences between these two groups (Figure 3B). Monocle3-based pseudotime analysis further showed that tumour cells followed a global trajectory from early neural progenitor cell-like (NPC-like) and oligodendrocyte progenitor cell-like (OPC-like) states towards terminal astrocyte-like (AC-like) and MES-like states (Figure 3C–E), consistent with the previously described PN–MES state transition [47].

Notably, key enzymes involved in AA metabolism, including *PLA2G4A*, *PTGS1*, *PTGS2*, *ALOX5*, and *CYP2J2*, displayed dynamic expression changes along this transition trajectory, as shown in the heatmap (Figure 3F). These findings suggest that AA metabolism-related transcriptional reprogramming is closely coupled with PN–MES-related malignant-state evolution in GBM. To contextualise AA pathway changes within the broader linoleic acid/PUFA-processing pathway, upstream PUFA-related genes, including *CD36*, *FADS1*, *FADS2*, and *ELOVL5*, were also examined (Figure 3F). In addition, we added a supplementary transcriptional phenotype analysis showing that AAMGs-high MES-like cells were associated with MES/EMT, migration/motility, matrix invasion/ECM remodelling, membrane-property remodelling, lipotoxicity/ferroptosis adaptation, hypoxia/NF-κB inflammatory activation, stemness/plasticity, therapy resistance, and survival/apoptosis-resistance signatures (Supplementary Figure S1).

### 3.3. Cell–Cell Communication Analysis Identifies the PPIA–BSG Axis as a Candidate Mediator of High AA Metabolism Mesenchymal Niches

**Figure 4:**
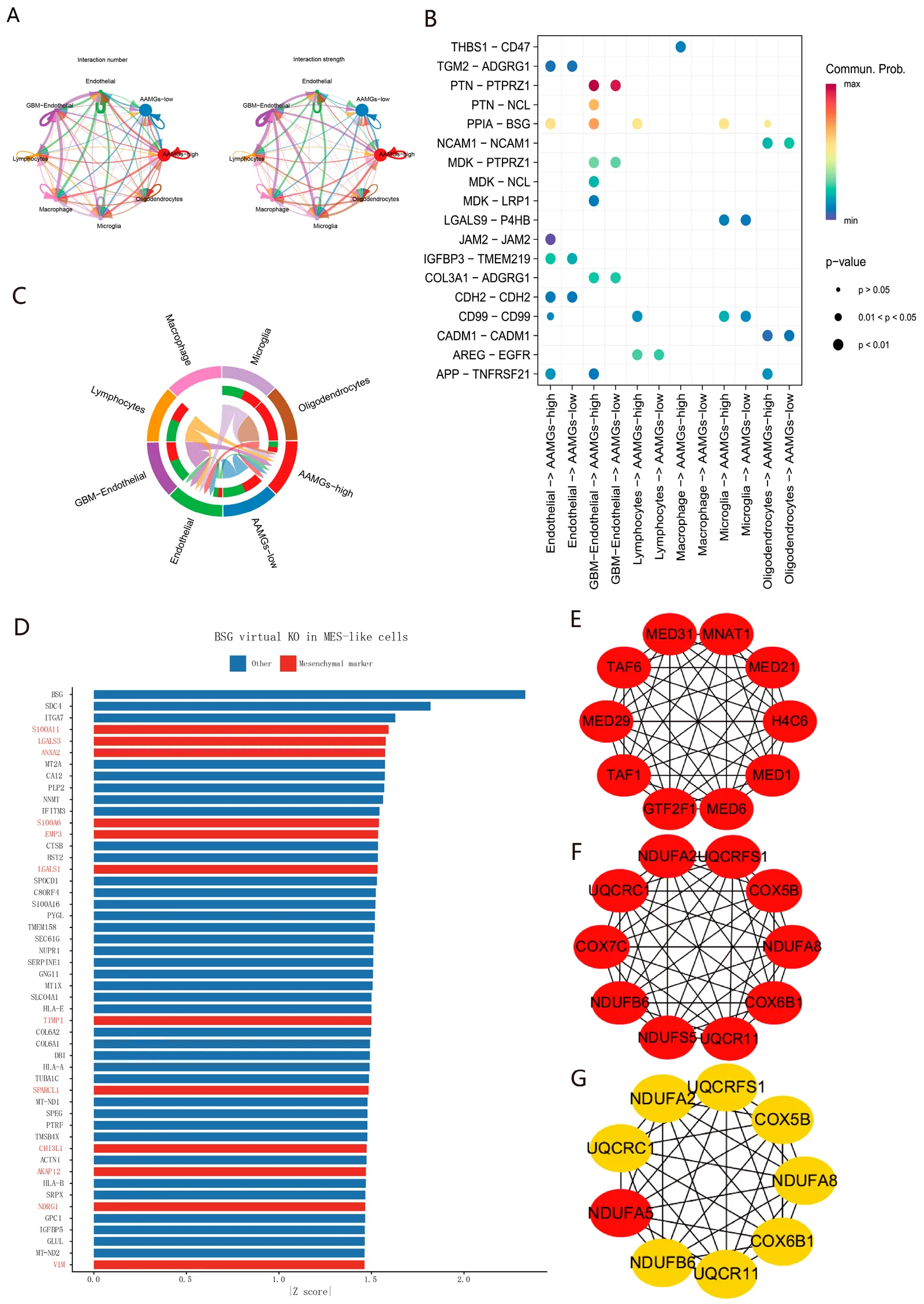
Cell–cell communication associated with the high arachidonic acid metabolic state and *BSG* perturbation analysis. (**A**) Overall communication networks inferred by CellChat between AAMGs-high or AAMGs-low malignant cells and cells in the tumour microenvironment; the panel on the left depicts the number of interactions, whereas the one on the right illustrates the corresponding interaction strength. (**B**) Bubble plot showing representative ligand–receptor interactions from different sender cell types toward AAMGs-high or AAMGs-low malignant cells; dot colour indicates communication probability, and dot size indicates statistical significance. *PPIA*-*BSG* signalling was mainly detected in AAMGs-high-associated communication. (**C**) Chord diagram further displaying communication relationships among AAMGs-high or AAMGs-low malignant cells and endothelial cells, GBM-associated endothelial cells, lymphocytes, macrophages, microglia, and oligodendrocytes. (**D**) Top 25 downregulated genes after scTenifoldKnk-based virtual *BSG* knockout in the MES-like malignant-cell subset; red indicates mesenchymal-related genes, and blue indicates other genes. (**E**–**G**) Protein–protein interaction networks and core module analysis based on *BSG* perturbation-associated genes, showing that transcriptional regulation and mitochondrial metabolism-related modules may contribute to *BSG*-associated regulation of malignant cell states.

CellChat analysis revealed that AAMGs-high tumour cells displayed more active communication networks with endothelial cells, macrophages, and microglia. Among the detected ligand–receptor interactions, *PPIA*–*BSG* signalling was consistently identified only in the AAMGs-high group, whereas comparable communication activity was not observed in the AAMGs-low group (Figure 4A–C). This finding suggests that the *BSG*-associated signalling axis may be closely linked to tumour–microenvironment interactions under a high AA metabolism state.

To further evaluate the regulatory relevance of *BSG*, we performed scTenifoldKnk-based virtual knockout of *BSG*. After *BSG* perturbation in MES-like malignant cells, the inferred regulatory network showed changes in multiple MES- and invasion-related genes and functional modules (Figure 4D), indicating that *BSG* may be associated with mesenchymal-like transcriptional features and microenvironmental interactions in AAMGs-high tumour cells.

A protein–protein interaction network was then constructed via STRING using genes perturbed after *BSG* virtual knockout. The resulting network exhibited a clear modular architecture. Subsequent Cytoscape-based analysis identified several core modules and hub nodes (Figure 4E–G). These modules were mainly involved in transcriptional regulation and mitochondrial metabolism-related processes, further suggesting that the regulation of AAMGs-high tumour cell states and their microenvironmental interactions may be mediated, at least in part, by *BSG*.

### 3.4. Spatial Transcriptomic Mapping Shows Co-Localisation of AAMGs-High Tumour Cells with MES-like Regions

To validate these findings at the spatial level, we mapped the single-cell reference atlas onto GBM spatial transcriptomic sections. Cell2location-based deconvolution revealed marked heterogeneity in cellular composition and metabolic niches within tumour tissues.

**Figure 5:**
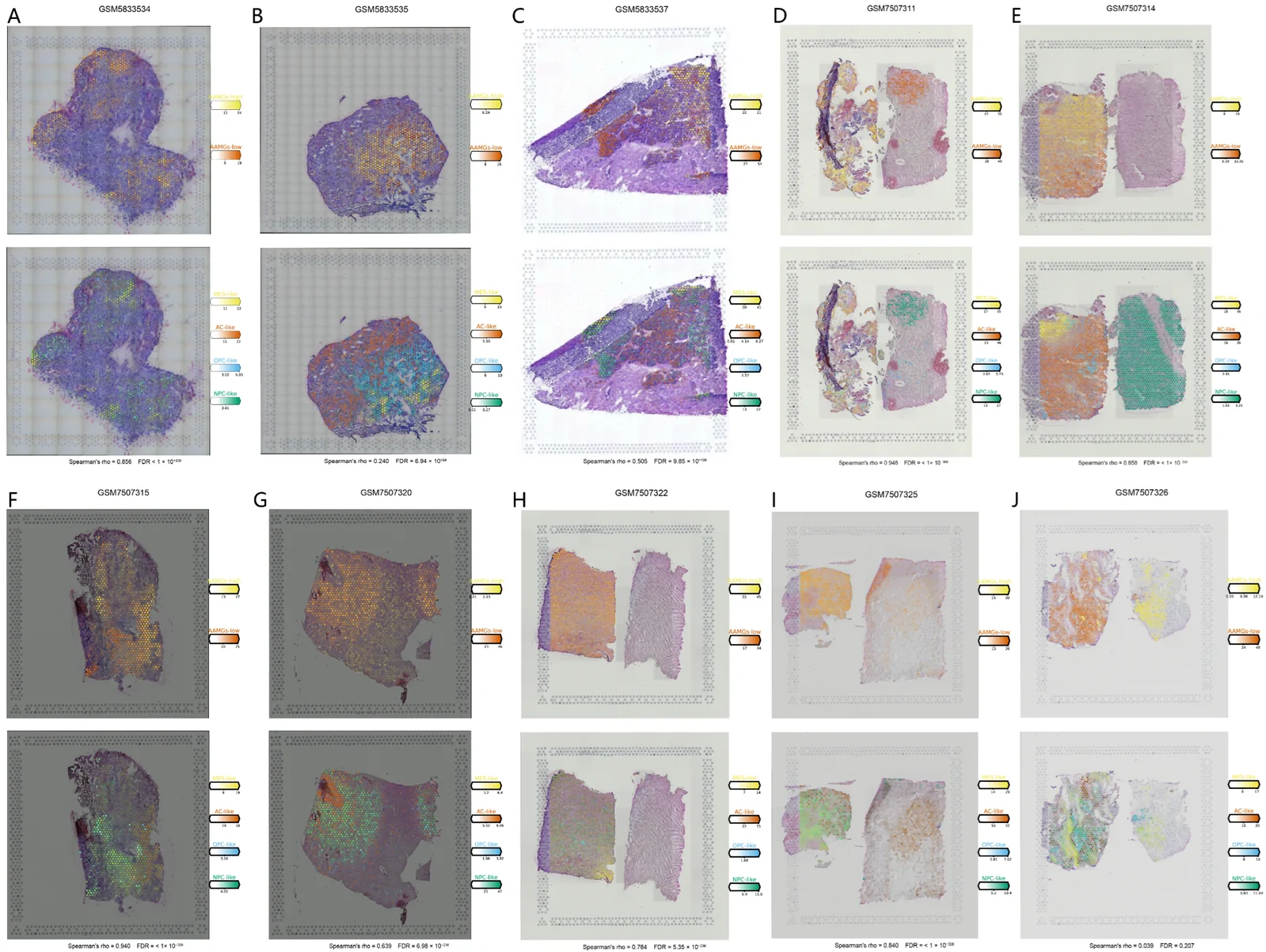
Spatial transcriptomic analysis shows heterogeneous spatial distributions of AAMGs-high/AAMGs-low malignant cells and MES-like, AC-like, OPC-like, and NPC-like cell states across ten GBM samples, with AAMGs-high regions co-localising with MES-like-enriched areas in most samples, suggesting a close association between arachidonic acid metabolic activation and mesenchymal-like malignant tumour niches. (**A**) Spatial distributions in sample GSM5833534 (Spearman’s rho = 0.856); (**B**) spatial distributions in sample GSM5833535 (Spearman’s rho = 0.240); (**C**) spatial distributions in sample GSM5833537 (Spearman’s rho = 0.505); (**D**) spatial distributions in sample GSM7507311 (Spearman’s rho = 0.948); (**E**) spatial distributions in sample GSM7507314 (Spearman’s rho = 0.858); (**F**) spatial distributions in sample GSM7507315 (Spearman’s rho = 0.940); (**G**) spatial distributions in sample GSM7507320 (Spearman’s rho = 0.639); (**H**) spatial distributions in sample GSM7507322 (Spearman’s rho = 0.764); (**I**) spatial distributions in sample GSM7507325 (Spearman’s rho = 0.840); and (**J**) spatial distributions in sample GSM7507326 (Spearman’s rho = 0.039).

Across most sections, regions enriched for AAMGs-high tumour cells demonstrated a strong positive spatial association with MES-like cell distribution and displayed evident co-localisation. In contrast, regions with relatively low AA metabolic activity were more frequently associated with enrichment of NPC-like or OPC-like cells (Figure 5A–J). These results indicate that AA metabolic activation is not a diffuse event across GBM tissues, but rather represents a spatially organised feature co-localised with mesenchymal-like tumour regions.

### 3.5. Spatial Analysis Links the PPIA–BSG-Associated Niche to LOX-Branch Activation and Inflammatory–Hypoxic Programmes

**Figure 6:**
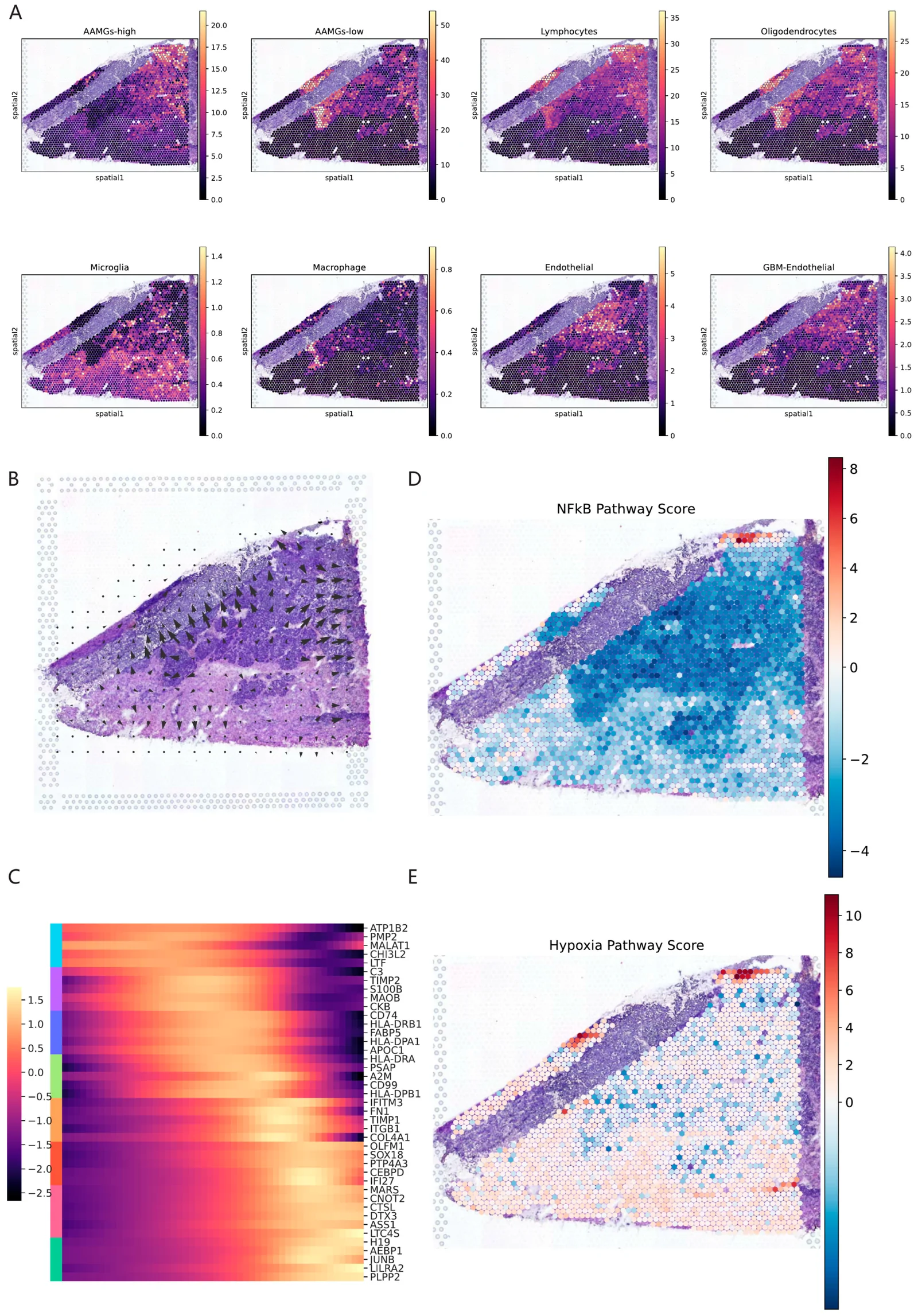
Spatial transcriptomic analysis reveals coupling between *PPIA*–*BSG*-associated niches and inflammatory-hypoxic programs. (**A**) Spatial distributions of AAMGs-high and AAMGs-low malignant cells, as well as lymphocytes, oligodendrocytes, microglia, macrophages, endothelial cells, and GBM-associated endothelial cells in the GSM5833537 sample. (**B**) COMMOT-inferred direction and intensity of *PPIA*–*BSG* spatial communication. (**C**) Heatmap of differentially expressed genes associated with *PPIA*–*BSG* spatial communication, showing expression changes in genes related to arachidonic acid metabolism and microenvironmental remodelling across spatial regions. (**D**) Spatial distribution of NF-κB pathway activity in the tissue section. (**E**) Spatial distribution of hypoxia pathway activity in the tissue section. Together, these results indicate that *PPIA*–*BSG*-associated communication is enriched in AAMGs-high malignant cells and adjacent microenvironmental regions, forming a high-metabolic mesenchymal-like malignant niche coupled with LOX-branch arachidonic acid metabolic remodelling, inflammatory activation, and hypoxic signalling.

Given the relatively clear spatial stratification of tumour cells in the GSM5833537 sample, we further applied COMMOT to characterise *PPIA*–*BSG*-mediated communication in this section (Figure 6A). *PPIA* and *BSG* were markedly enriched in specific tumour regions (Figure 6B). COMMOT-dependent differential gene expression analysis further showed that, among AA metabolism-related molecules associated with this communication programme, *ALOX5*, *LTC4S*, *GGT5*, and *PTGDS* were upregulated, whereas *AKR1C3* was downregulated (Figure 6C). These changes suggest that, within specific spatial niches, the *PPIA*–*BSG* axis may be associated with activation of the LOX branch and partial remodelling of prostaglandin metabolism, thereby shaping a local microenvironment characterised by high AA metabolic activity.

We next inferred pathway activity across spatial spots using PROGENy. Using Moran’s I, we observed significant spatial autocorrelation in NF-κB, hypoxia, AA metabolism, and *BSG* expression, with the strongest spatial clustering observed for NF-κB and hypoxia (NF-κB: I = 0.579; hypoxia: I = 0.286; both FDR = 0; Figure 6D,E). A significant positive correlation between NF-κB and hypoxia was further identified through correlation analysis, whereas their relationships with *BSG* expression and AA metabolism were more complex. Together, these results suggest that *PPIA*–*BSG*-associated niches are coupled with inflammatory and hypoxic transcriptional programmes and may contribute to AA metabolic reprogramming in GBM.

### 3.6. Arachidonic Acid Metabolism-Related Gene Signatures Enable Robust Prognostic Stratification in GBM

**Figure 7:**
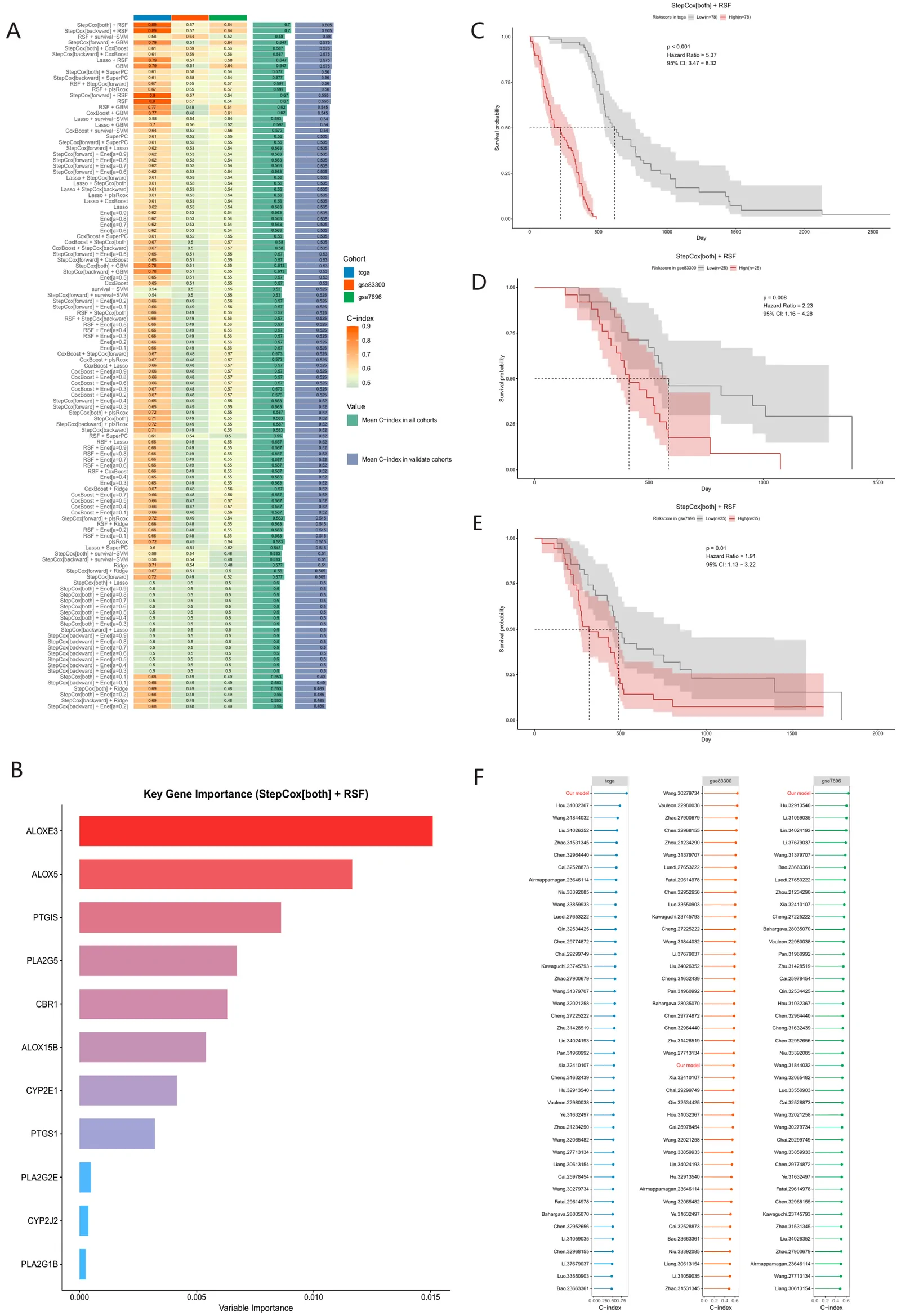
Construction and validation of a machine learning prognostic model based on arachidonic acid metabolism-related genes. (**A**) C-index comparison of 101 machine learning combinations using TCGA-GBM as the training cohort and GSE83300 and GSE7696 as validation cohorts, leading to the selection of the StepCox[both] + RSF model. (**B**) Variable importance of the 11 core genes included in the final model, including *ALOXE3*, *ALOX5*, *PTGIS*, *PLA2G5*, *CBR1*, *ALOX15B*, *CYP2E1*, *PTGS1*, *PLA2G2E*, *CYP2J2*, and *PLA2G1B*. (**C**–**E**) Kaplan–Meier survival curves for high- and low-risk groups in the TCGA, GSE83300, and GSE7696 cohorts, showing poorer overall survival in the high-risk group. Horizontal and vertical dashed lines indicate the 50% survival probability and the corresponding median survival time, respectively. (**F**) C-index comparison between the present model and published GBM prognostic models across different cohorts, indicating favourable risk stratification ability and external validation performance.

To evaluate the prognostic relevance of AA metabolism at the patient level, we used 63 AAMGs as candidate features and developed 101 machine-learning survival models in bulk RNA-sequencing cohorts, using TCGA-GBM as the training set and GSE7696 and GSE83300 as validation sets. Based on the average concordance index, the StepCox[both] + RSF model achieved the best overall performance, with a mean concordance index (C-index) of approximately 0.700 in the training cohort and 0.605 in the validation cohorts (Figure 7A).

Variable-importance analysis identified 11 core genes in the final model: *ALOXE3*, *ALOX5*, *PTGIS*, *PLA2G5*, *CBR1*, *ALOX15B*, *CYP2E1*, *PTGS1*, *PLA2G2E*, *CYP2J2*, and *PLA2G1B* (Figure 7B). Based on Kaplan–Meier survival analysis, patients classified as high-risk showed markedly poorer outcomes compared to the low-risk group across both cohorts, with significant divergence of survival curves (*p* < 0.05; Figure 7C–E). The model robustly stratified patients into high- and low-risk groups and outperformed previously reported GBM prognostic models in TCGA and GSE7696. However, in GSE83300, the model did not show a clear advantage over existing models (Figure 7F).

### 3.7. Virtual Screening Identifies Venetoclax as a Candidate Compound Targeting BSG

**Figure 8:**
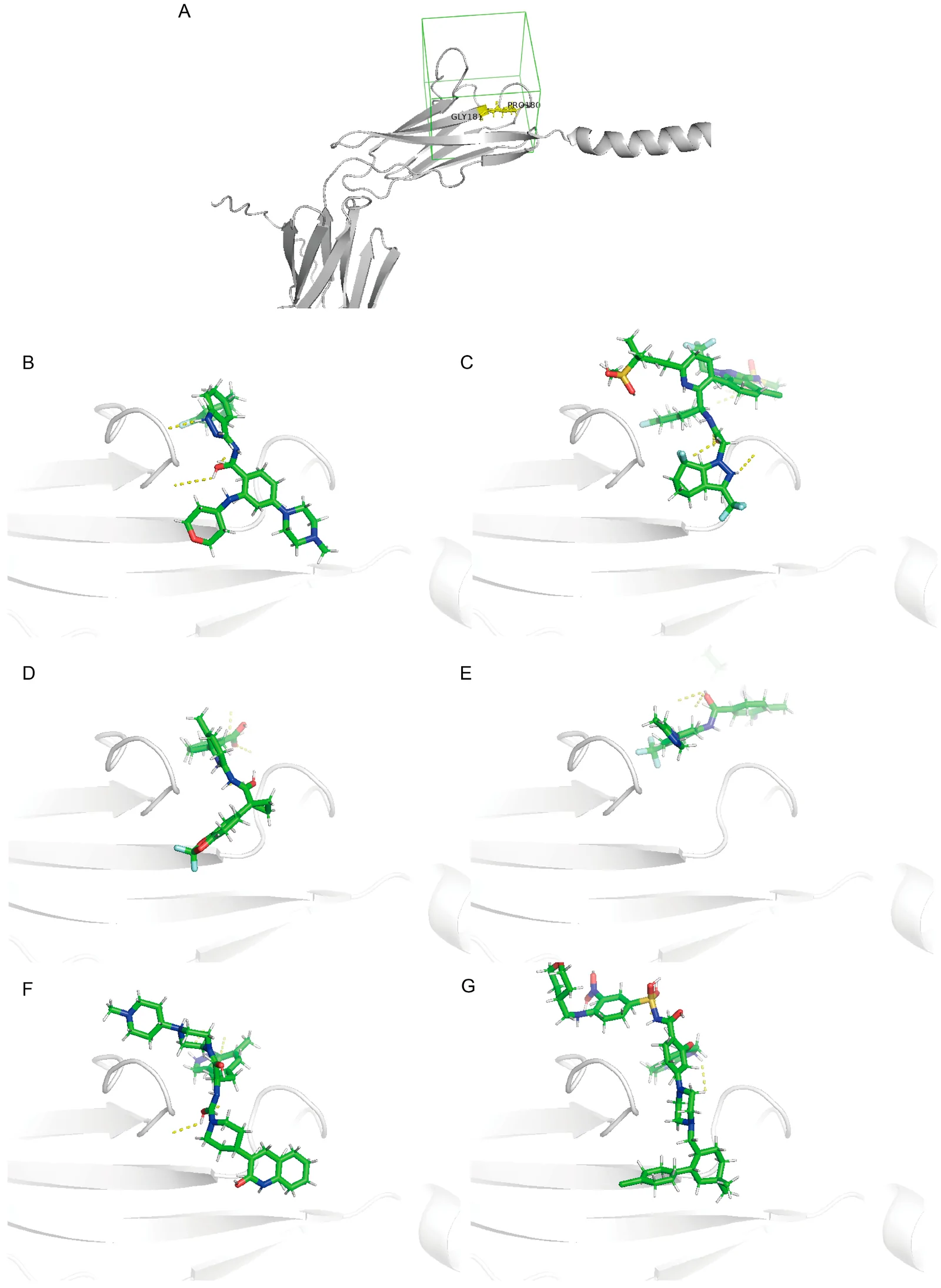
Structure-based virtual screening of *BSG* and docking conformations of candidate small molecules. (**A**) AlphaFold-predicted *BSG* protein structure and the molecular docking search region, with the green box indicating the Vina docking box. (**B**–**G**) Docking conformations of six representative candidate compounds within the *BSG* binding pocket, including Entrectinib, Lenacapavir, Lumacaftor, Nilotinib, Vazegepant, and Venetoclax, with the green stick structures representing the small-molecule ligands. Overall, the results show that Venetoclax can fit into the *BSG* binding region, providing a structural basis for subsequent molecular dynamics simulations and in vitro functional validation.

Using the AlphaFold-predicted *BSG* protein structure, we defined a docking pocket around the binding region reported in the literature and performed virtual screening with the TargetMol U.S. Food and Drug Administration (FDA)-approved small-molecule drug library (L4200). The Vina docking box was centred at coordinates x = 2.73, y = 20.75, and z = 8.27, with each box dimension set to 20 Å (Figure 8A).

Based on binding affinity and docking-pose plausibility, six representative candidate compounds were selected: Entrectinib, Lenacapavir, Lumacaftor, Nilotinib, Vazegepant, and Venetoclax (Figure 8B–G). Considering binding energy, docking conformation, pocket compatibility, and feasibility for downstream validation, Venetoclax was ultimately selected for further investigation. Its best-ranked docking pose showed a binding affinity of −7.7 kcal mol^−1^ (Table 1). Conformational visualisation indicated that Venetoclax could be accommodated within the *BSG* pocket, providing a structural basis for subsequent molecular dynamics analysis and in vitro functional validation.

### 3.8. Molecular Dynamics Simulations Support Stable Binding Between Venetoclax and BSG

**Figure 9:**
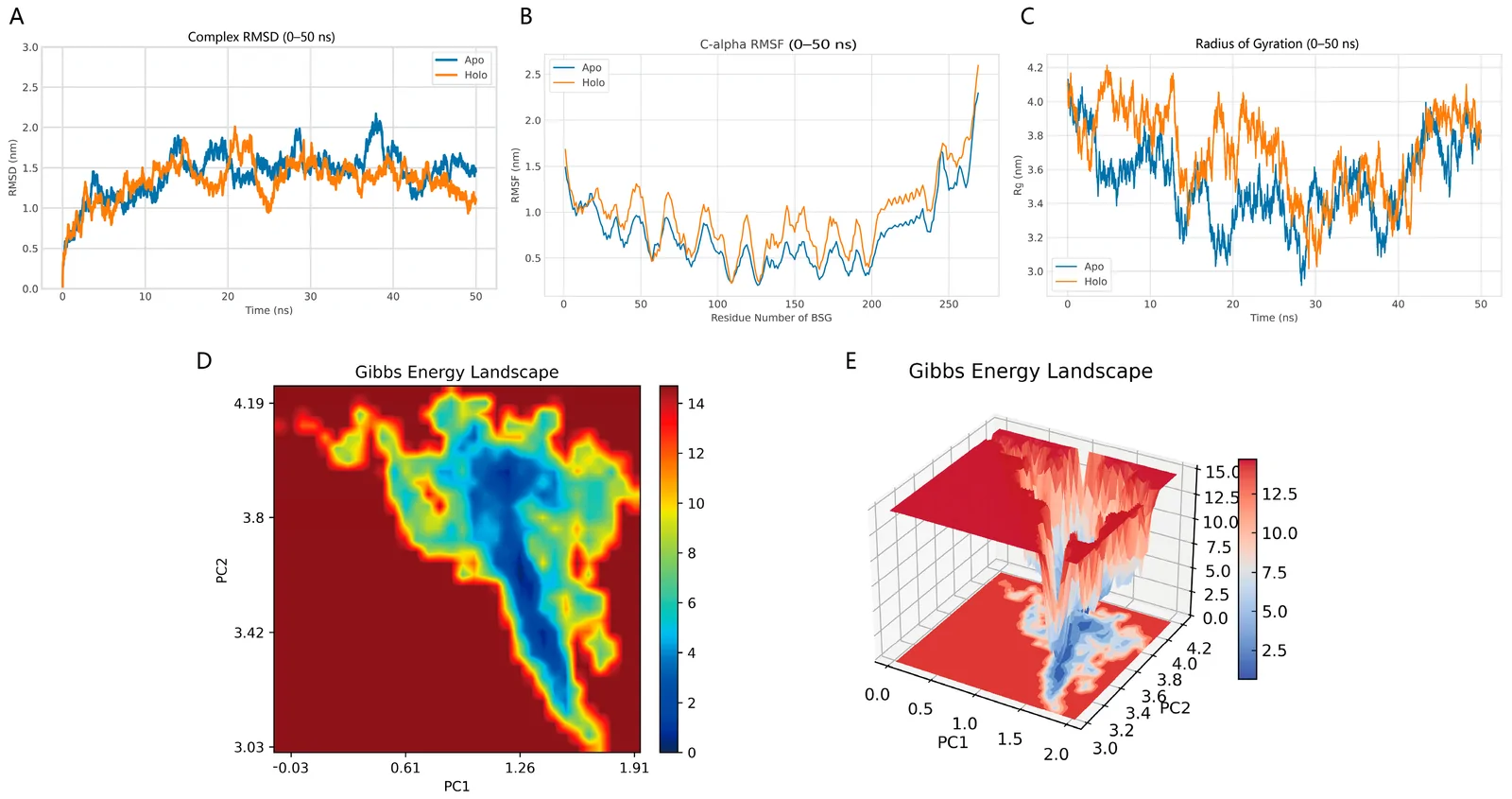
Molecular dynamics simulation analysis of the *BSG*–Venetoclax complex. (**A**) RMSD changes of the apo *BSG* and holo *BSG*–Venetoclax systems during the 0–50 ns simulation used to evaluate overall conformational stability. (**B**) C-alpha RMSF curves showing local flexibility differences across *BSG* residues. (**C**) Time-dependent radius of gyration reflecting dynamic fluctuations in protein compactness. (**D**,**E**) Two-dimensional and three-dimensional Gibbs free energy landscapes constructed from principal components PC1 and PC2, showing a major low-energy conformational region for the *BSG*–Venetoclax complex and supporting its relatively stable binding state during the simulation.

To assess the stability of the docking result, we performed molecular dynamics simulations of both apo *BSG* and the holo *BSG*–Venetoclax complex. After an initial conformational adjustment during the first approximately 10 ns, the RMSD of both systems gradually reached a relatively stable plateau (Figure 9A). The overall RMSD of the complex was maintained mainly within approximately 1.2–1.5 nm, with only transient increases during limited intervals, suggesting that the *BSG*–Venetoclax complex retained favourable global structural stability within the analysed time window.

RMSF analysis showed that structural fluctuations were mainly concentrated at the protein termini and selected flexible loop regions, whereas most core regions exhibited relatively limited fluctuations (Figure 9B). Radius of gyration analysis indicated moderate conformational contraction and expansion during the simulation, without persistent structural destabilisation (Figure 9C). Free-energy landscape analysis identified a dominant low-energy basin and showed no marked conformational transition (Figure 9D,E), supporting relatively stable thermodynamic behaviour of the complex. These findings should nevertheless be interpreted as computational evidence and require further validation through biochemical binding assays and functional experiments.

### 3.9. Venetoclax Suppresses GBM Cell Viability and Attenuates Mesenchymal-like Features

**Figure 10:**
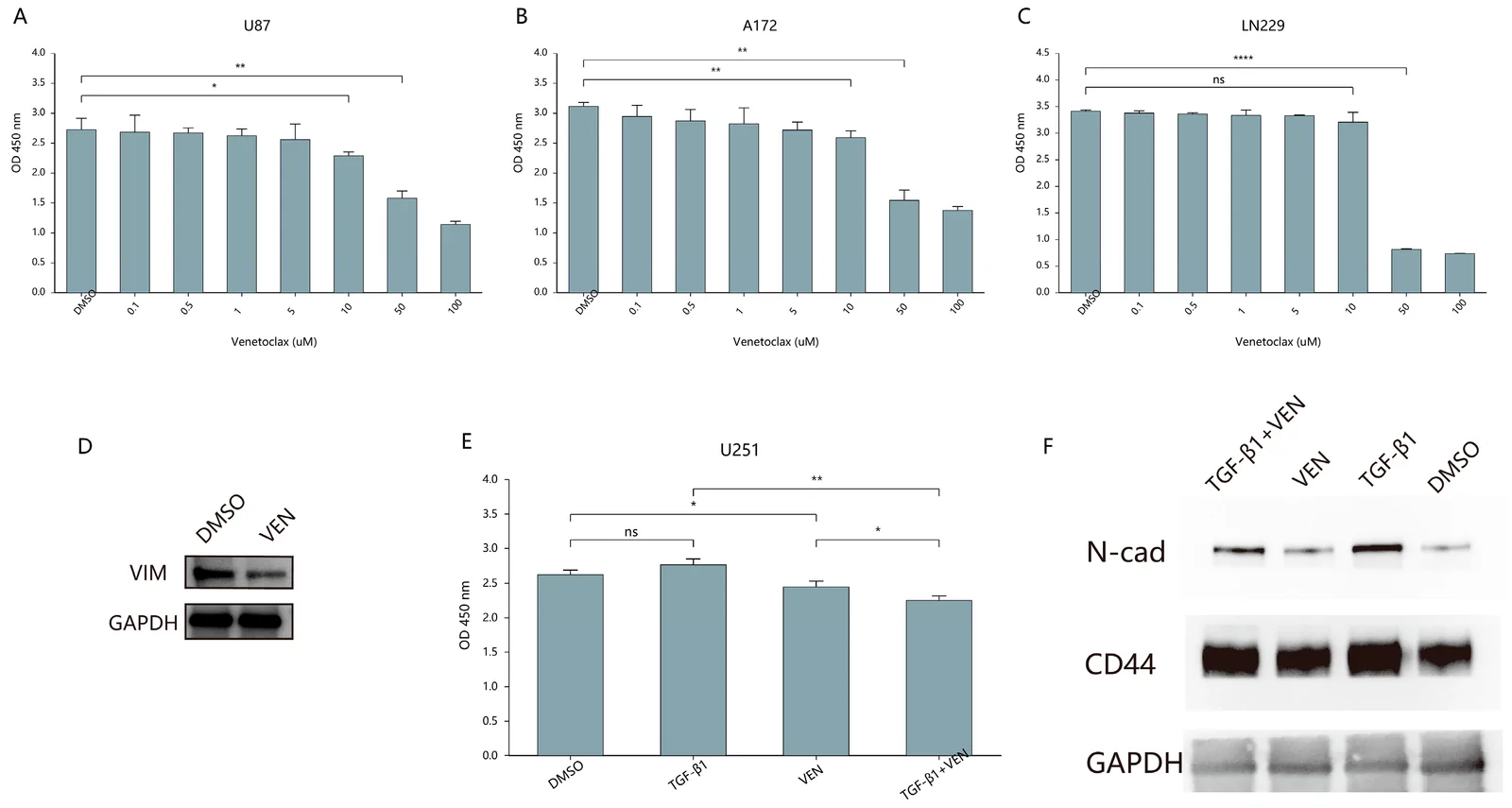
Effects of Venetoclax on GBM cell viability and mesenchymal-like marker expression. (**A**–**C**) CCK-8 assays showing changes in cell viability of U87, A172, and LN229 cells after treatment with different concentrations of Venetoclax. Venetoclax showed dose-dependent inhibitory effects on U87 and A172 cells and significantly reduced the viability of all three GBM cell lines at higher concentrations. (**D**) Western blotting showed decreased VIM protein expression in U87 cells after treatment with 10 μM Venetoclax for 48 h, suggesting that Venetoclax may attenuate mesenchymal-like features in GBM cells. (**E**) CCK-8 assay showing U251 cell viability after 48 h of treatment with DMSO, TGF-β1, Venetoclax (VEN), or TGF-β1 plus VEN. TGF-β1 alone produced a non-significant numerical increase relative to DMSO; VEN was lower than DMSO, TGF-β1 plus VEN was lower than TGF-β1, and TGF-β1 plus VEN was also lower than VEN. (**F**) Western blot analysis of N-cadherin and CD44 expression in U251 cells treated with DMSO, TGF-β1, VEN, or TGF-β1 plus VEN. ns, not significant; * *p* < 0.05; ** *p* < 0.01; **** *p* < 0.0001.

The effect of Venetoclax on GBM cell viability was further evaluated using CCK-8 assays. Venetoclax exerted dose-dependent inhibitory effects on U87 and A172 cells, with a mild but significant reduction in cell viability already observed at 10 μM. In contrast, LN229 cells showed no significant change at 10 μM. At concentrations of 50 μM and above, cell viability was markedly reduced in all three GBM cell lines (Figure 10A–C).

In U87 cells, 10 μM Venetoclax treatment resulted in a modest but significant decrease in cell viability compared with the DMSO control group, as reflected by the 450 nm optical density values (DMSO control = 2.726 ± 0.191; Venetoclax 10 μM = 2.291 ± 0.061; *p* = 0.0197; Figure 10A). In A172 cells, 10 μM Venetoclax similarly reduced cell viability relative to the DMSO control (DMSO control = 3.117 ± 0.065; Venetoclax 10 μM = 2.594 ± 0.112; *p* = 0.0022; Figure 10B). In LN229 cells, however, 10 μM Venetoclax did not induce a statistically significant change in cell viability (DMSO control = 3.412 ± 0.022; Venetoclax 10 μM = 3.209 ± 0.183; *p* = 0.1282; Figure 10C). Based on these results, 10 μM was selected as the concentration for subsequent mechanistic experiments.

Western blot analysis further showed that treatment with 10 μM Venetoclax for 48 h reduced the expression of the epithelial–mesenchymal transition (EMT)-related marker VIM in U87 cells (Figure 10D). Together with the single-cell and spatial transcriptomic findings, these results suggest that Venetoclax may interfere with *BSG*-associated signalling and thereby attenuate mesenchymal-like and inflammatory activation states in GBM cells.

To directly examine U251 cell viability under the four treatment conditions, CCK-8 assays were performed after 48 h. The TGF-β1-only group showed a modest numerical increase relative to DMSO, but the difference was not statistically significant (2.763 ± 0.086 versus 2.624 ± 0.060; *p* = 0.0832). Venetoclax reduced the CCK-8 signal in the absence of TGF-β1 (VEN versus DMSO: 2.446 ± 0.086 versus 2.624 ± 0.060; *p* = 0.0422) and during TGF-β1 exposure (TGF-β1 + VEN versus TGF-β1: 2.251 ± 0.065 versus 2.763 ± 0.086; *p* = 0.0012). The TGF-β1 + VEN group also showed a lower CCK-8 signal than VEN alone (*p* = 0.0360; Figure 10E). Thus, the Venetoclax-associated reduction in the U251 CCK-8 readout was retained in the TGF-β1-conditioned setting; the pairwise difference between the two Venetoclax-containing groups was not interpreted as evidence of pharmacological synergy.

In U251 cells, TGF-β1 increased PN–MES-related markers, including N-cadherin and CD44, whereas co-treatment with Venetoclax attenuated these marker changes (Figure 10F). These data suggest that Venetoclax may influence PN–MES-related marker changes.

## 4. Discussion

Glioblastoma (GBM) is characterised by marked cell-state plasticity, metabolic heterogeneity, and microenvironmental complexity, which together form the biological basis of its aggressive behaviour and therapeutic resistance. With the development of single-cell and spatial omics technologies, GBM research has progressively shifted from a focus on tumour-intrinsic molecular alterations alone towards a systems-level understanding of tumour cell state transitions, metabolic reprogramming, and their interactions with the tumour microenvironment [48].

The proneural-to-mesenchymal (PN–MES) transition in GBM is not a single molecular event, but rather a cell-state reprogramming process jointly driven by tumour-intrinsic transcriptional regulation, therapeutic pressure, and microenvironmental cues [3]. Verhaak et al. first established transcriptome-based molecular subtypes of GBM, laying the foundation for the study of proneural and mesenchymal phenotypes [2]. Bhat et al. further demonstrated that a subset of proneural glioma stem-like cells can transition towards a mesenchymal state in a TNF-α/NF-κB-dependent manner, accompanied by CD44 enrichment and enhanced radioresistance, suggesting an important role for microenvironmental components such as macrophages and microglia in promoting this process [4]. Subsequent single-cell studies have further conceptualised GBM as a continuum of plastic cellular states, including NPC-like, OPC-like, AC-like, and MES-like states, rather than as a collection of discrete and static subtypes. These findings indicate that the PN–MES transition is essentially a dynamic shift along a malignant cell-state axis under the combined influence of genetic background and external stimuli.

In this study, we show that arachidonic acid (AA) metabolic reprogramming is not a diffuse background alteration in GBM, but rather a defining component of mesenchymal-associated malignant niches with clear spatial organisation. Moreover, the *PPIA*–*BSG* signalling axis may serve as a candidate interface linking a high AA metabolism state, microenvironmental communication, and the coupling of inflammatory and hypoxic transcriptional programmes.

First, our findings suggest that AA metabolic reprogramming may constitute an integral component of malignant-state evolution in GBM, rather than a passive by-product of tumour metabolism. By integrating four public GBM single-cell transcriptomic cohorts, we found that AA metabolism module scores differed markedly across malignant states, with the highest scores observed in MES-like cells and relatively lower scores in NPC-like and OPC-like states. This state-dependent pattern suggests that activation of AA metabolism may occur in parallel with the PN–MES-related state transition and may contribute to the acquisition of more invasive and treatment-resistant phenotypes. The supplementary phenotype-signature analysis further suggests that the AAMGs-high MES-like state is linked at the transcriptomic level to cell-state programs relevant to migration, extracellular matrix invasion, membrane remodelling, lipotoxicity/ferroptosis adaptation, and therapy resistance, providing a biological rationale for why AA pathway activation may be favoured in inflammatory, hypoxic, and mesenchymal niches [49].

This observation is consistent with previous evidence linking AA metabolism to mesenchymal differentiation and therapeutic resistance in GBM [13,14]. Bhat et al. reported that mesenchymal differentiation in GBM is accompanied by NF-κB activation and increased radioresistance, and that COX-2 expression is associated with mesenchymal features and poor radiotherapy response [50,51,52]. Notably, more recent studies suggest that, compared with global changes in the COX axis, the lipoxygenase branch may be more directly involved in shaping malignant GBM niches. The ALOX5/5-HETE axis is upregulated in glioma and can promote M2-like polarisation of glioma-associated microglia/macrophages and PD-L1 expression, thereby contributing to an immunosuppressive microenvironment [53]. Conversely, inhibition of ALOX5 or other LOX-branch components can attenuate GBM cell growth, migration, and treatment tolerance [16,54]. Together with the close association of the MES state with inflammatory amplification, myeloid cell enrichment, and hypoxia, these findings support the possibility that AA metabolism, particularly LOX-branch activation, provides an important metabolic basis for the establishment and maintenance of mesenchymal-like GBM states [5,55].

These results are also in line with the broader understanding of GBM mesenchymalisation. Previous studies have shown that GBM cells can transition from proneural towards mesenchymal states under diverse microenvironmental stimuli, and that the MES subtype is typically associated with poorer prognosis, greater invasiveness, and more pronounced immunosuppression [2,3,4,56]. This process is known to involve immune microenvironmental cells, transcription factor networks, inflammatory mediators, and environmental stress [9,57,58,59,60]. The distinction of the present study is that it further links this process to lipid metabolic reprogramming, suggesting that high AA metabolism may represent a metabolic foundation of mesenchymal-associated malignant niches in GBM [13].

Beyond state-dependent metabolic features, we identified the *PPIA*–*BSG* axis as a candidate mechanism connecting the high AA metabolism state with microenvironmental crosstalk. Biologically, *BSG*, also known as CD147, is a transmembrane glycoprotein associated with tumour invasion, anti-apoptotic capacity, and resistance to radiotherapy and chemotherapy [61,62,63,64,65,66]. *PPIA* is one of its canonical ligands and can mediate extracellular signal transduction in local inflammatory and stress-related contexts [67,68]. In this study, the *PPIA*–*BSG* axis appears to function as a key connecting node. On the one hand, it is associated with malignant tumour cells exhibiting a high AA metabolism state; on the other hand, it links these cells with a microenvironmental network composed of microglia, lymphocytes, oligodendrocytes, endothelial cells, and GBM-associated endothelial cells. In this way, metabolic reprogramming, mesenchymal-like transcriptional programmes, and inflammatory signalling may be integrated within the same local tumour niche.

Another important contribution of this study is the extension of single-cell-level inference to the spatial tissue context. By mapping the single-cell reference atlas onto GBM spatial transcriptomic sections, we found that regions enriched in AAMGs-high tumour cells substantially overlapped with MES-like regions. AA metabolic remodelling in GBM also showed apparent branch specificity, characterised by LOX-branch activation and remodelling of COX-branch metabolism. These findings indicate that AA metabolic activation is not diffusely distributed across GBM tissues, but instead represents a regional feature with clear spatial boundaries, consistent with the concept of a localised malignant metabolic niche.

Further COMMOT-based analysis of *PPIA*–*BSG* communication showed that *PPIA* and *BSG* were synchronously enriched in AAMGs-high regions. These results suggest that AA metabolic reprogramming is not only cell-state dependent, but also spatially organised. In other words, activation of distinct AA metabolic branches is not randomly distributed, but is coupled with MES-like cell enrichment, enhanced *PPIA*–*BSG* communication, and local microenvironmental remodelling.

Notably, PROGENy-based spatial pathway activity inference further showed significant spatial autocorrelation of NF-κB, hypoxia, AA metabolism, and *BSG* expression, with the most prominent clustering observed for NF-κB and hypoxia. Together with the correlation analysis, these findings suggest that AAMGs-high/*BSG*-high regions are unlikely to be defined by lipid metabolic activation alone. Instead, they may represent composite niches jointly shaped by inflammation, hypoxia, and metabolic reprogramming. Given that hypoxia can drive lipid metabolic adaptation and promote invasion-associated transcriptional programmes, while NF-κB is a central hub connecting inflammatory stimulation with tumour progression, our results suggest that *PPIA*–*BSG*-associated niches may promote a local shift towards a more invasive and therapy-resistant mesenchymal-like state by coupling inflammatory and hypoxic programmes with abnormal AA metabolism. Compared with a simple description of metabolic differences, this spatial evidence provides more direct tissue-level support for the potential coupling among metabolic state, cell–cell communication, and niche architecture.

At the translational level, our study further indicates that AA metabolism-related features may have patient-level prognostic value. Using 63 AAMGs, we constructed and compared 101 machine-learning survival models in the TCGA-GBM training cohort and the GSE7696 and GSE83300 validation cohorts, ultimately identifying an optimal model composed of 11 core genes. Kaplan–Meier survival curves indicated that patients classified into the high-risk subgroup exhibited markedly worse overall survival in both the training and validation cohorts, suggesting that AA metabolism-related programmes are not only present in local malignant states at the single-cell and spatial levels, but can also be captured as a stable prognostic feature at the whole-patient level.

The risk stratification capacity of this model may not depend on the effect of any single metabolic molecule. Rather, it probably reflects integrated biological information across multiple dimensions, including tumour cell state, local microenvironmental interactions, and spatial heterogeneity. In this sense, AA metabolism-related features may provide a more integrative indicator of GBM aggressiveness than single-molecule biomarkers.

However, the performance of the model varied across external validation cohorts, particularly in GSE83300, where it did not show a clear advantage over previously reported models. This finding indicates that, although AA metabolism-related prognostic modelling has potential clinical utility, its generalisability across different platforms, populations, and molecular backgrounds requires further validation in larger and preferably prospective cohorts. In addition, bulk RNA-based models cannot fully distinguish signals derived from malignant cells from those originating in the microenvironment [69]. Future integration with spatial subtyping, single-cell deconvolution, and digital pathology features may improve the robustness and biological interpretability of such models.

In addition to mechanistic inference and risk assessment, we preliminarily explored the druggability of the *BSG*-associated signalling axis through drug repurposing [70,71]. We selected an FDA-approved drug library for virtual screening, because drug repurposing offers practical advantages for translational research in GBM [72,73,74]. Compared with de novo drug development, approved drugs generally have clearer safety profiles, pharmacokinetic information, and clinical use experience, which may shorten the translational timeline and reduce development risk. Previous reviews have also suggested that drug repurposing represents one of the more feasible strategies for therapeutic development in GBM.

Based on virtual screening of an FDA-approved small-molecule library and the AlphaFold-predicted *BSG* structure, we identified Venetoclax as a potential candidate compound. Venetoclax showed a favourable docking score with *BSG*, and molecular dynamics analysis further suggested a certain degree of conformational stability of the *BSG*–Venetoclax complex. In vitro experiments showed that higher concentrations of Venetoclax significantly suppressed the viability of U87, A172, and LN229 cells. Western blot analysis further showed decreased expression of mesenchymal markers such as VIM after Venetoclax treatment, suggesting that Venetoclax may partially attenuate the mesenchymal-like phenotype of GBM cells. These results are directionally consistent with the single-cell and spatial transcriptomic analyses, suggesting that Venetoclax may suppress mesenchymalisation and inflammatory activation states in GBM cells by interfering with *BSG*-associated signalling networks.

Venetoclax reduced the CCK-8 signal in U251 cells both in the absence of and during exposure to TGF-β1. This observation is consistent with a recent U251 study reporting concentration-dependent antiproliferative activity of ABT-199, with an IC50 of 15.67 μM after 72 h [75]. Because tetrazolium-based assays such as CCK-8 primarily measure cellular metabolic activity, the present result does not by itself distinguish reduced proliferation or metabolic activity from cell death [76]. The modest, non-significant increase in the TGF-β1-only group is consistent with an independent U87/U251 study in which TGF-β1 produced a slight, non-significant increase in proliferation while reducing apoptosis [77]. The lower CCK-8 signal in the TGF-β1 plus Venetoclax group than in the Venetoclax-only group further indicates that the TGF-β1-conditioned context did not blunt the observed response; however, this pairwise comparison does not establish synergy without a formal interaction analysis. Together with the attenuation of TGF-β1-induced N-cadherin and CD44 changes, these findings provide supportive evidence from a TGF-β1-conditioned, PN–MES-related U251 model, but do not establish that Venetoclax specifically blocks PN–MES transition.

Nevertheless, these findings should be interpreted cautiously. First, although the in vitro inhibitory effect of Venetoclax is consistent with the direction of the single-cell and spatial transcriptomic inferences, the current data only suggest that Venetoclax may affect *BSG*-associated niches; they do not establish Venetoclax as a direct or specific *BSG* inhibitor. Second, Venetoclax has a complex and well-established pharmacological background, and its effects on cell viability and EMT- or inflammation-related markers may not be mediated solely through *BSG*. Therefore, at the current stage, Venetoclax should be regarded as a candidate intervention molecule with potential exploratory value rather than a validated *BSG*-targeting drug. Future studies incorporating biophysical binding assays, *BSG*-dependent rescue experiments, and in vivo models are required to clarify the directness and specificity of Venetoclax against the *BSG*-associated signalling axis. In addition, the U251 experiment was added only as a PN-like cell-line substitute because we could not perform paired PN and MES patient-derived GSC experiments under the current experimental conditions.

This study has several limitations. First, the single-cell, spatial transcriptomic, and bulk transcriptomic cohorts were mainly obtained from public databases. Differences in sample origin, sequencing platform, and clinical background may have introduced retrospective bias. Second, the *PPIA*–*BSG* axis was identified primarily through cell–cell communication inference, spatial co-localisation, and virtual knockout analysis, and therefore still lacks direct support from ligand–receptor blocking assays and biochemical binding experiments. This axis may not simply mark a high AA metabolism state, but may instead function as an interface that integrates metabolic state, inflammatory stimulation, and mesenchymal transcriptional programmes within the same local niche.

Third, the relationship between AA metabolism and PN–MES-related state transition remains mainly associative. Its causal role requires further validation through metabolic flux analysis, perturbation of key metabolic enzymes, and rescue experiments. Fourth, the fluctuating performance of the prognostic model across external validation cohorts indicates that its clinical transferability needs further evaluation. Fifth, the mechanism of action of Venetoclax remains incompletely defined, particularly with regard to the need to exclude effects mediated by targets other than *BSG*. Future studies integrating metabolomics, spatial multi-omics, and in vivo functional experiments will be necessary to clarify the formation mechanism and therapeutic plasticity of AA metabolism-associated malignant niches in GBM. More specifically, mRNA expression of AAMGs, *CD36*, *FADS1*, *FADS2*, and *ELOVL5* cannot be assumed to equal lipid uptake, enzymatic flux, intracellular AA abundance, or eicosanoid production; these mechanisms require mass spectrometry lipidomics, isotope-tracing flux analysis, and functional rescue assays.

In summary, this study demonstrates that AA metabolic reprogramming is an important feature of mesenchymal-associated malignant niches in GBM and may be coupled with inflammatory and hypoxic programmes through *PPIA*–*BSG*-mediated cell–cell communication. These findings extend our understanding of metabolic heterogeneity and microenvironmental crosstalk in GBM, and suggest that AA metabolism-related features and the *BSG*-associated signalling axis may represent promising directions for risk stratification and targeted intervention.

## 5. Conclusions

This study shows that arachidonic acid metabolic reprogramming plays an important role in GBM progression by coupling with PN–MES state transition, inflammatory and hypoxic programmes, and *PPIA*–*BSG*-mediated cell–cell communication. Activation of AA metabolism is not only closely associated with the MES-like phenotype and poor patient prognosis, but may also promote invasive evolution and therapeutic resistance of GBM cells by shaping mesenchymal-associated malignant niches. Therefore, the present data support a transcriptomic and spatial association between AA pathway gene expression and PN–MES-related malignant states, but do not by themselves prove altered lipid metabolic flux.

Further analysis suggests that the *BSG*-associated signalling axis may represent a key node linking metabolic dysregulation, microenvironmental crosstalk, and tumour cell state transition. Venetoclax, identified as a potential candidate compound, showed inhibitory effects on GBM cell viability and attenuated mesenchymal-like features in vitro. A deeper understanding of how AA metabolic reprogramming and the *PPIA*–*BSG* axis influence these processes may improve our knowledge of GBM pathogenesis and reveal new opportunities for risk stratification and therapeutic intervention. Continued investigation will be essential to clarify the underlying mechanisms, validate potential targeting strategies, and ultimately improve clinical outcomes for patients with GBM.

## Figures and Tables

**Table 1:** Molecular docking results of Venetoclax (DrugBank ID: DB11581) with *BSG* (UniProtKB isoform ID: P35613-2; AlphaFold3-predicted structure).

Mode	Affinity	RMSD_lb	RMSD_ub
1	−7.7	0	0
2	−7.31	4.013	12.264
3	−7.194	4.027	12.472
4	−7.152	6.042	11.789
5	−7.128	4.156	6.985
6	−7.087	3.818	6.393
7	−7.07	1.556	2.238
8	−7.059	1.91	2.558
9	−6.837	3.932	13.096

## Data Availability

The data presented in this study are derived from publicly available repositories. Single-cell RNA-sequencing (scRNA-seq) data were obtained from NCBI GEO (https://www.ncbi.nlm.nih.gov/geo/; accessed on 27 March 2026) under accession numbers GSE103224, GSE138794, GSE139448, and GSE230389. Spatial transcriptomics (stRNA-seq) data were obtained from NCBI GEO under accession numbers GSE194329 and GSE235672. Bulk RNA-sequencing (bulk RNA-seq) data were obtained from NCBI GEO under accession numbers GSE83300 and GSE7696. TCGA glioblastoma data were obtained from UCSC Xena (https://xenabrowser.net/datapages/; accessed on 16 March 2026).

## Supplementary Materials

The following supporting information can be downloaded at: https://www.mdpi.com/article/10.3390/biomedicines14081738/s1, Figure S1: AAMGs-High Property Enrichment in MES-like Cells; Table S1: Clinical and Molecular Characteristics of scRNA-seq Samples [78,79,80,81].

## Abbreviations

The following abbreviations are used in this manuscript:

AA	Arachidonic acid
AAMGs	Arachidonic acid metabolism-related genes
MES	Mesenchymal
AC	Astrocyte
OPC	Oligodendrocyte precursor cell
NPC	Neural progenitor cell
PN	Proneural
GBM	Glioblastoma
*PPIA*	Peptidylprolyl isomerase A
*BSG*	Basigin
