# Supplementary material for: Integrative Single-Cell and Spatial Transcriptomic Analyses Link Arachidonic Acid Metabolic Reprogramming to Proneural-to-Mesenchymal State Transition in Glioblastoma
Source: Biomedicines. 2026 Aug 1;14(8):1738. doi: 10.3390/biomedicines14081738 (PMC13510037; doi:10.3390/biomedicines14081738)
Supplement: Supplementary file 1 [file biomedicines-14-01738-s001.zip › Supplementary/Supplementary Figure S1.pdf]

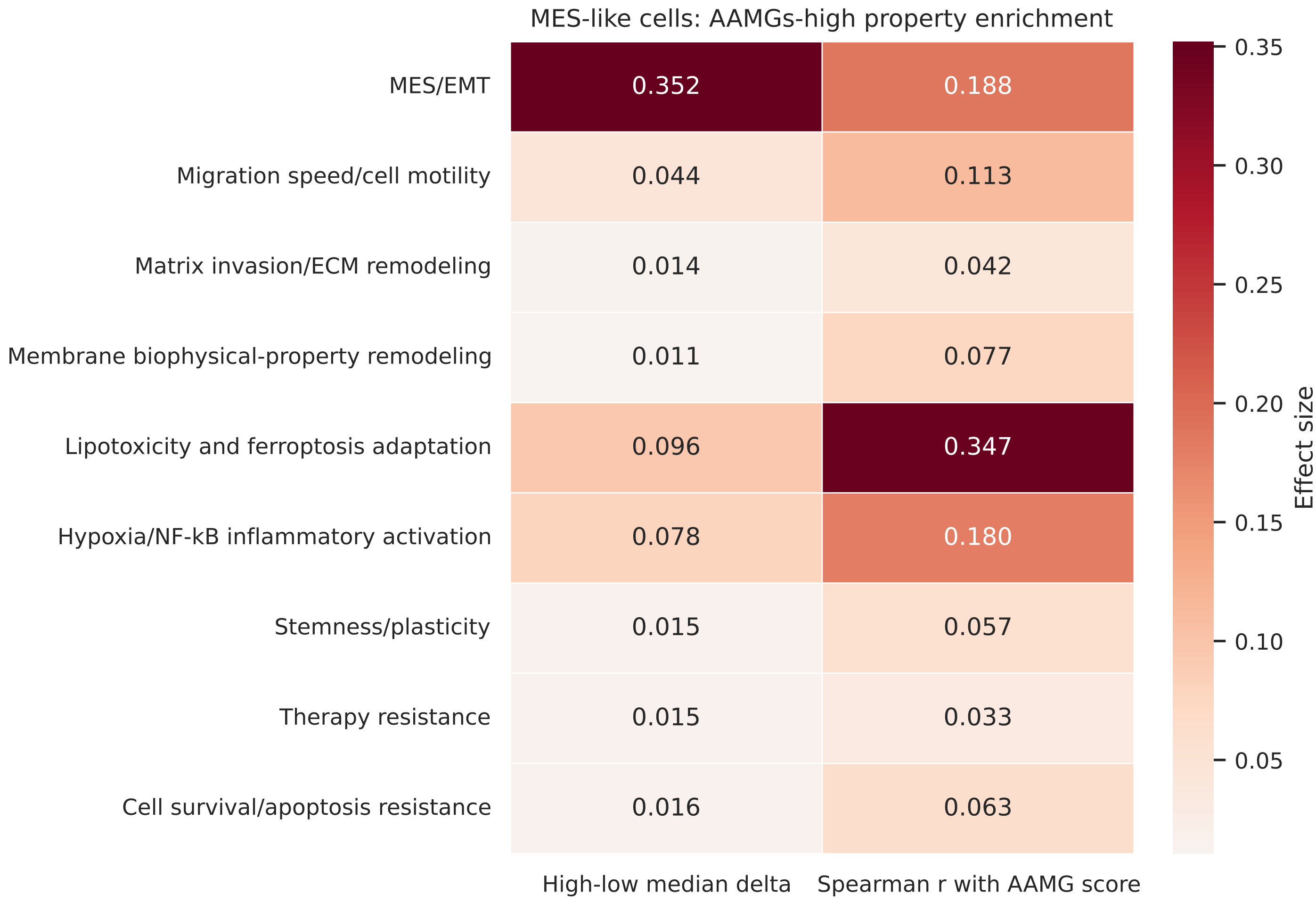
MES-like cells: AAMGs-high property enrichment
MES/EMT
Migration speed/cell motility
Matrix invasion/ECM remodeling
Membrane biophysical-property remodeling
Lipotoxicity and ferroptosis adaptation
Hypoxia/NF-kB inflammatory activation
Stemness/plasticity
Therapy resistance
Cell survival/apoptosis resistance
0.352
0.188
0.044
0.113
0.014
0.042
0.011
0.077
0.096
0.347
0.078
0.180
0.015
0.057
0.015
0.033
0.016
0.063
High-low median delta
Spearman r with AAMG score
Effect size
0.35
0.30
0.25
0.20
0.15
0.10
0.05
